# Prevalence of cardiac fibrosis and infiltrative cardiomyopathy in patients with advanced conduction system disease

**DOI:** 10.1002/joa3.70109

**Published:** 2025-07-06

**Authors:** Jeremy William, Haider Muthana, Joseph Hogarty, Andrew Taylor, James L. Hare, Justin Mariani, Hitesh Patel, Geoff Wong, Dion Stub, David M. Kaye, Sandeep Prabhu, Peter M. Kistler, Aleksandr Voskoboinik

**Affiliations:** ^1^ The Alfred Hospital Melbourne Australia; ^2^ Monash University Melbourne Australia; ^3^ The Baker Heart and Diabetes Research Institute Melbourne Australia; ^4^ Royal Melbourne Hospital Melbourne Australia

**Keywords:** cardiac MRI, conduction disease, fibrosis, infiltrative cardiomyopathy

## Abstract

**Background:**

Conduction system disease may represent an early manifestation of underlying structural heart disease, including infiltrative disorders. Timely diagnosis of underlying cardiomyopathy has significant implications for clinical management, guiding both disease‐modifying medical therapy and decisions around device implantation.

**Objective:**

We sought to investigate the utility of cardiac magnetic resonance imaging (CMR) in patients with conduction system disease and preserved LV function on echocardiography.

**Methods:**

We evaluated all patients undergoing CMR between 2005 and 2023 at our institution for the investigation of advanced conduction system disease (complete heart block, Mobitz II block, or bifascicular block). We excluded patients with known systolic heart failure (LVEF<50%) prior to CMR. We evaluated the prevalence of CMR‐detected myocardial fibrosis and infiltrative cardiomyopathy in this cohort.

**Results:**

One hundred nineteen patients were identified (mean age 49 ± 15 years, 52% male). Complete heart block was the most common indication (50%), followed by bifascicular block (27%) and Mobitz II block (23%). Mean LVEF on echocardiography prior to CMR was 60.0 ± 3.1%. CMR‐detected late gadolinium enhancement (LGE) was present in 32/119 patients (26.9%). Cardiac sarcoid was the most common final diagnosis (*n* = 19, 16%), of whom only five (26%) had known extracardiac sarcoid prior to CMR. Cardiac fibrosis was observed in a similar proportion of patients across the three subtypes of conduction disease studied (*p* = 0.47).

**Conclusion:**

Cardiac fibrosis is present in a substantial proportion of patients undergoing CMR for the investigation of conduction disease, even when LV function appears preserved on echocardiography. Cardiac MRI may be an important adjunctive tool for the investigation of conduction disease, particularly in younger patients.

## Graphical abstract

Prevalence of cardiac fibrosis and sarcoidosis in patients with advanced conduction system disease.

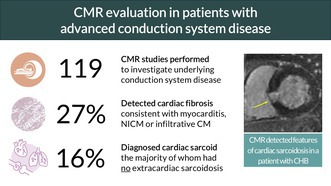

## 1 INTRODUCTION

Disease of the cardiac conduction system can produce a spectrum of patterns on the 12‐lead electrocardiogram (EKG), ranging from isolated hemifascicular block to complete atrioventricular (AV) block or ventricular standstill.^1^ While these disorders are most often related to age‐related degeneration of the conduction system or overt structural heart disease,^2^ there exists a subset of patients with unexplained conduction system disease, in whom these traditional etiologies are absent.

In recent years, there has been growing recognition that advanced conduction system disease in younger adults may represent an early manifestation of an underlying cardiomyopathy (CMP).^3^ In this setting, initial evaluation with standard transthoracic echocardiography may not detect subtle features of early‐stage structural heart disease. Conversely, advanced cardiac imaging modalities such as cardiac magnetic resonance imaging (CMR) can provide myocardial tissue characterization to identify the presence of fibrosis or edema as hallmarks of an underlying CMP. CMR‐detected cardiac sarcoidosis (CS) has been reported in 11%–30% of middle‐aged adults presenting with complete heart block, although screening protocols and cohort ages were heterogeneous in these reports.^4^, ^5^ Conduction system disease is also commonly seen in cardiac amyloidosis, with up to 33% of patients featuring interventricular conduction delay and 10% featuring high‐degree AV block.^6^

Understanding the prevalence of infiltrative CMP in younger patients with unexplained conduction disease has important clinical implications. Timely diagnosis of an underlying infiltrative CMP can lead to earlier access to targeted medical therapy and may guide decision‐making around cardiac implantable electronic device (CIED) therapy.

## 2 METHODS

We performed a retrospective review of all patients undergoing CMR for the evaluation of unexplained conduction disease at a single center in Melbourne, Australia. Ethics approval for the study was sought from the local institutional ethics committee (Alfred Health Ethics Committee, approval number 22/147). All CMR imaging reports between January 2010 and December 2023 were reviewed. We selected patients in whom the indication for CMR was unexplained advanced conduction system disease. This included defined (1) bifascicular block pattern (either right bundle branch block plus hemifascicular or left bundle branch block), (2) type 2 secondary degree AV block (Mobitz II block), or (3) third degree AV block (complete heart block; CHB). We included patients with and without CIEDs at the time of CMR. We excluded patients with isolated right bundle branch block or hemifascicular block patterns. We also excluded patients with known LV systolic dysfunction, defined as left ventricular ejection fraction (LVEF) <50% on TTE preceding CMR and patients with LV wall thickness >15 mm, as these patients had a competing CMR indication for the evaluation of CMP.

### 2.1 CMR evaluation

Cardiac magnetic resonance imaging was conducted on all patients using a clinical 1.5‐T scanner (Signa HD 1.5‐T, GE Healthcare). Initial cine CMR sequences were obtained in three standard long‐axis views (four‐chamber, three‐chamber, and two‐chamber) as well as in short‐axis slices (basal, mid, and apical). Left ventricular volume and function were calculated using either biplane analysis or a contiguous short‐axis cine stack. LV scar was assessed using late gadolinium enhancement imaging conducted in long‐ and short‐axis views 10 min after administering a 20 mmol bolus of either gadobutrol (0.2 mmol/kg, Gadovist, Bayer) or gadopentetic acid (0.2 mmol/kg body weight, Magnevist, Schering, Germany). The presence or absence of LV scar was determined as a binary outcome using visual thresholding on a contiguous short‐axis LGE stack (if available) or three short‐axis slices through the basal, mid, and apical LV using CMR42 (Circle Cardiovascular Imaging Inc., Calgary, Canada).

### 2.2 Additional workup after CMR

For patients in whom CMR features were suggestive of infiltrative cardiomyopathy, we reviewed the results of subsequent downstream investigations including endomyocardial biopsy (EMB), extracardiac biopsies, and fluorodeoxyglucose positron emission tomography (FDG‐PET). We then recorded the final clinical diagnosis as informed by all available investigations. Cases classified as CS met criteria for either “definitive” or “highly probable” sarcoidosis in accordance with the 2024 American Heart Association Scientific Statement for the diagnosis and management of cardiac sarcoidosis.^7^

### 2.3 Statistical analysis

Normally distributed continuous data were summarized as mean ± standard deviation and analyzed by the Student's *t*‐test. Skewed continuous data are presented as median with interquartile range and were analyzed using the Mann–Whitney *U* test. Categorical variables are presented as frequency (%) and were analyzed using the chi‐squared test for cohorts >50 patients or the Fisher exact test for cohorts <50 patients. A two‐sided *p* value of <0.05 was considered statistically significant. Statistical analyses were performed using SPSS version 27 (IBM, Armonk, NY, USA). A *p*‐value <0.05 was considered statistically significant.

## 3 RESULTS

### 3.1 Cohort characteristics

One hundred nineteen patients who underwent CMR evaluation for unexplained conduction disease between 2010 and 2023 met the specified inclusion criteria and were incorporated into this study. Clinical characteristics are shown in Table 1. The mean cohort age was 49.0 ± 15.3 years, and 52.1% of patients were male. The most frequent indication for CMR evaluation was CHB (60 patients, 50.4%), followed by bifascicular block (32 patients, 26.9%) and Mobitz II AV block (27 patients, 22.7%). Forty‐three patients (36.1%) had an existing permanent pacemaker at the time of CMR evaluation; no patients had an existing implantable cardioverter defibrillator (ICD). Mean LVEF on TTE prior to CMR evaluation was 60.0 ± 3.1%. 18.5% of patients in this study had an existing diagnosis of extracardiac sarcoidosis at the time of CMR referral.

**TABLE 1:** Cohort characteristics.

	All patients (*n* = 119)	LGE positive (*n* = 32)	LGE negative (*n* = 87)	*p*‐value
Clinical features				
Age (mean ± SD)	49.0 ± 15.3	57.6 ± 12.9	45.9 ± 14.9	**<0.001**
Male (*n*, %)	62 (52.1%)	18 (56.3%)	44 (50.6%)	0.58
BMI (kg/m^2^, mean ± SD)	27.3 ± 5.8	28.1 ± 7.4	27 ± 5.2	0.38
Hypertension (*n*, %)	19 (16.0%)	8 (25.0%)	11 (12.6%)	0.10
Diabetes (*n*, %)	7 (14.0%)	3 (9.4%)	4 (4.6%)	0.32
Current smoker (*n*, %)	13 (10.9%)	3 (9.4%)	10 (11.5%)	0.74
LVEF on TTE (%, mean ± SD)	60.0 ± 3.1	60.2 ± 3.1	59.9 ± 3.1	0.70
Clinical presentation				
Syncope	29 (24.3%)	8 (25.0%)	21 (24.1%)	0.92
Presyncope	29 (24.3%)	9 (28.1%)	20 (23.0%)	0.56
Asymptomatic/incidental	61 (51.2%)	15 (46.9%)	46 (52.9%)	0.56
Distal conduction disease type				
Complete heart block (*n*, %)	60 (50.4%)	18 (56.3%)	42 (48.3%)	0.44
Mobitz II block (*n*, %)	27 (22.7%)	6 (18.8%)	21 (24.1%)	0.53
Bifascicular block (*n*, %)	32 (26.9%)	8 (25%)	24 (27.6%)	0.77
Extracardiac sarcoidosis				
Known extracardiac sarcoid at time of CMR (*n*, %)	22 (18.5%)	6 (18.8%)	16 (18.4%)	0.96
Pulmonary sarcoidosis (*n*, %)	18 (15.1%)	4 (12.5%)	14 (16.1%)	0.62
Cutaneous sarcoidosis (*n*, %)	3 (2.5%)	1 (3.1%)	2 (2.3%)	0.80
Lymph node sarcoidosis (*n*, %)	1 (0.8%)	1 (3.1%)	0 (0%)	N/A

*Note*: Bolding indicates statistical significance (*p* < .05).

Abbreviations: BMI, body mass index; CMR, cardiac magnetic resonance imaging; LVEF, left ventricular ejection fraction; SD, standard deviation; TTE, transthoracic echocardiogram.

### 3.2 CMR evaluation

The results of the CMR evaluation are shown in Table 2. Overall, CMR‐detected LGE suggestive of myocardial fibrosis was present in 32/119 patients (26.9%). LGE‐positive patients were older than LGE‐negative patients (57.6 ± 12.9 vs. 45.9 ± 14.9 years, *p* < 0.001). Other clinical characteristics were similar between the two groups (Table 1). The prevalence of LGE positivity was similar amongst patients with CHB, Mobitz II block, and bifascicular block (*p* = 0.65). With respect to CMR characteristics, LGE‐positive patients exhibited higher average LV mass index (64.1 ± 19.6 vs. 56.3 ± 16.2 g/m,^2^ *p* = 0.03) and higher average short tau inversion recovery (STIR) ratio (2.2 ± 0.6 vs. 1.9 ± 0.5, *p* = 0.02). A trend toward lower LVEF in LGE‐positive patients was observed (56.1 ± 8.5 vs. 59.5 ± 8.4, *p* = 0.05). Of note, 16 patients (13.4%) had LV dysfunction (LVEF <50%) detected on CMR that was not detected on TTE.

**TABLE 2:** CMR characteristics.

	All patients (*n* = 119)	LGE positive (*n* = 32)	LGE negative (*n* = 87)	*p*‐value
LVEDVi (mL/m^2^)	90 ± 20.4	89.2 ± 21.3	90.2 ± 20.2	0.80
LVEF (%)	58.6 ± 8.6	56.1 ± 8.5	59.5 ± 8.4	0.05
LVEF <50% (*n*, %)	16 (13.4%)	6 (18.8%)	10 (11.5%)	0.30
LVMI (g/m^2^)	56.9 ± 19.5	64.1 ± 19.6	56.3 ± 16.2	**0.03**
RV basal diameter (mm)	45.4 ± 7.7	46.7 ± 9.3	45.1 ± 7.4	0.56
RV FAC (%, mean)	48.6 ± 7.6	44.7 ± 6.7	47.7 ± 8	0.22
RA area (cm^2^)	21.5 ± 5.5	22.6 ± 6.4	21.1 ± 5.1	0.23
LAVI (mL/m^2^)	36.6 ± 10.3	39.1 ± 7.8	35.9 ± 10.8	0.36
Average STIR ratio	2.0 ± 0.5	2.2 ± 0.6	1.9 ± 0.5	**0.02**

*Note*: Bolding indicates statistical significance (*p* < .05).

Abbreviations: FAC, Fractional Area Change; LAVI, Left Atrial Volume Index; LVEF, Left Ventricular Ejection Fraction; LVEDVi, Left Ventricular End‐Diastolic Volume Index; LVMI, Left Ventricular Mass Index; RA, Right Atrial; RV, Right Ventricular; STIR, Short Tau Inversion Recovery.

Distribution patterns of detected LGE are described in Table 3. The most common distribution pattern was subepicardial or mid‐myocardial, present in 23 patients (71.9%), followed by transmural involvement in 6 patients (18.8%) and isolated subendocardial enhancement in 3 patients (9.4%). Interventricular septal (IVS) involvement was observed in 75.0% of cases, while nonseptal LGE was present in 50.0%. In this study, 68.8% of patients demonstrated LGE affecting two or more myocardial segments, while the remaining 31.2% had LGE confined to a single segment.

**TABLE 3:** Characterization of late gadolinium enhancement patterns.

	All LGE+ patients (*n* = 32)	Final diagnosis CS (*n* = 19)	Final diagnosis non‐CS (*n* = 13)	*p* value
Distribution of LGE				
Subepicardial/mid‐myocardial	23 (71.9%)	14 (73.7%)	9 (69.2%)	0.72
Subendocardial only	3 (9.4%)	0 (0%)	3 (23.1%)	0.06
Transmural	6 (18.8%)	4 (21.1%)	2 (15.4%)	1.0
Anatomical location				
Septal involvement	24 (75.0%)	14 (73.7%)	10 (76.9%)	1.0
Nonseptal involvement	16 (50.0%)	10 (52.6%)	6 (46.2%)	0.91
RV involvement	6 (18.8%)	5 (26.3%)	1 (7.7%)	0.36
LGE extent				
≥2 myocardial segments	22 (68.8%)	16 (84.2%)	6 (46.2%)	**0.04**
1 segment only	10 (31.2%)	3 (15.8%)	7 (53.8%)	**0.04**

*Note*: Bolding indicates statistical significance (*p* < .05).

Abbreviations: LGE, late gadolinium enhancement; RV, right ventricular.

**FIGURE 1:**
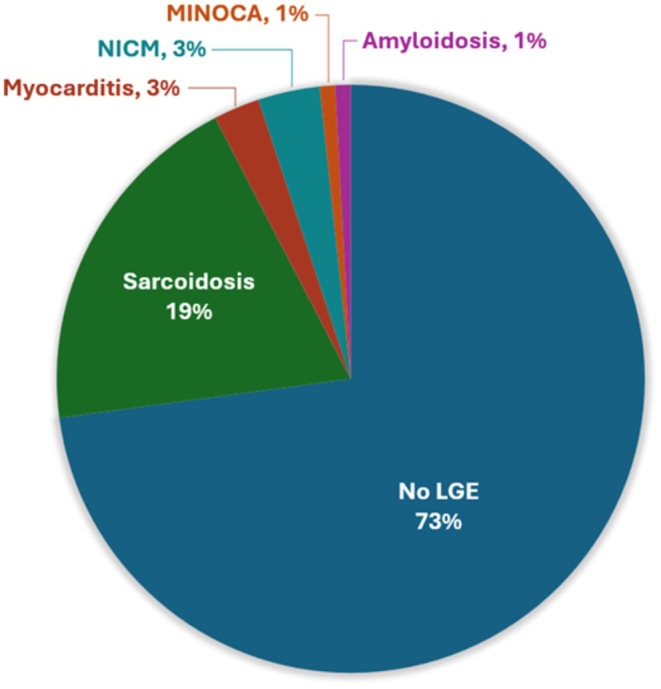
Diagnosis suggested by LGE pattern in patient's receiving CMR to evaluate advanced conduction system disease.

**FIGURE 2:**
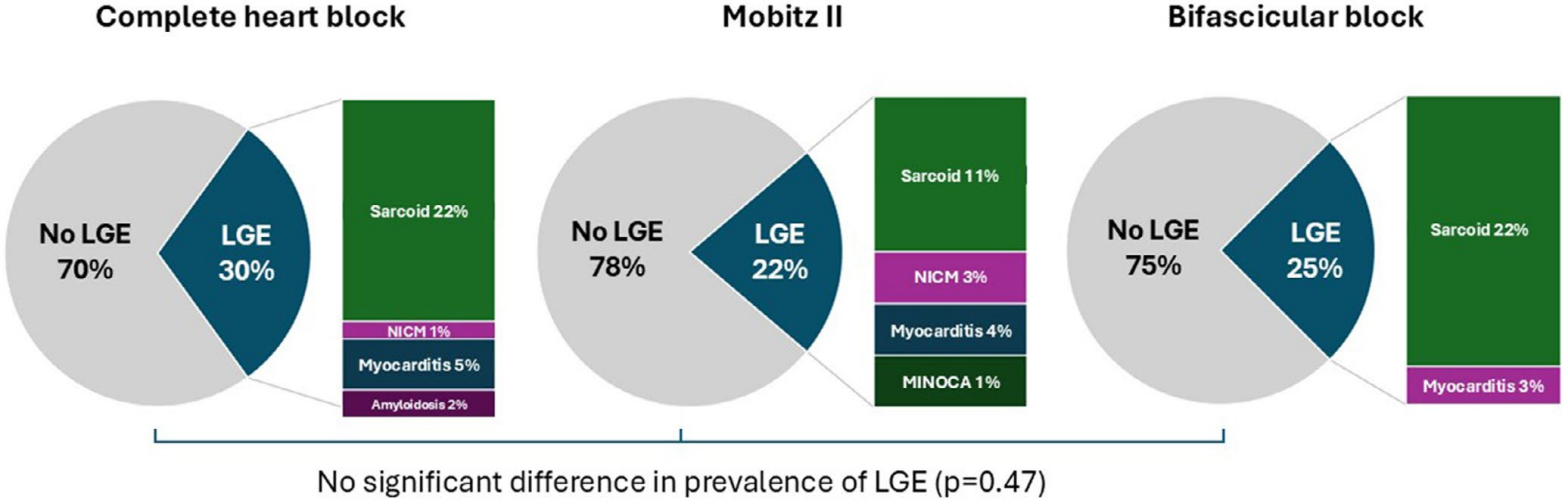
LGE pattern on CMR by conduction disease subtype.

Specific diagnoses suggested by the LGE pattern are shown in Figure 1. Patchy, dense, noncoronary distribution of LGE most consistent with CS was present in 23 patients (19.3%). Other CMR‐guided diagnoses included nonischemic CMP (3 patients, 2.5%), myocarditis (4 patients, 3.4%), previous myocardial infarction (1 patient, 0.84%), and cardiac amyloidosis (1 patient, 0.84%). A CMR diagnosis of CS was made in a similar proportion of patients across the three subtypes of conduction disease studied (*p* = 0.47, Figure 2).

### 3.3 Subsequent clinical progress

Table 4 details the subsequent investigations performed in 32 patients in whom CMR‐detected LGE. EMB was performed in 12/32 patients (37.5%) and revealed histological evidence of infiltrative or inflammatory CMP in 12 patients (CS in 9 patients, transthyretin (ATTR) cardiac amyloidosis in 1 patient, active lymphocytic myocarditis in 2 patients). PET scans were performed in 14/32 patients (43.8%), with myocardial fluorodeoxyglucose (FDG) avidity present in 9. Seven patients had a pattern consistent with active CS on PET scan, while two had features more consistent with lymphocytic myocarditis. Extracardiac biopsy was performed in 14/32 patients (43.8%), and histology revealed granulomatous changes consistent with sarcoidosis in 10 patients. Overall, 19 patients (16.0%) received a diagnosis of CS supported by histological data. Of these 19 patients, only 5 (26.0%) had a known diagnosis of extracardiac sarcoidosis at the time of CMR evaluation. Two additional patients were empirically diagnosed with probable CS on the basis of CMR and PET features without histologically proven sarcoidosis.

**TABLE 4:** Post‐CMR investigations of the 32 patients in whom CMR‐detected LGE.

32 patients with CMR‐detected LGE in the setting of advanced conduction disease
**Endomyocardial biopsy performed in 12 patients (37.5%)**
Cardiac sarcoidosis confirmed in 9 patients ATTR cardiac amyloidosis confirmed in 1 patient Lymphocytic myocarditis confirmed in 2 patients
**Extracardiac biopsy performed in 14 patients (43.8%)**
Granulomatous change suggestive of sarcoidosis confirmed in 10 patients
**FDG‐PET imaging performed in 14 patients (43.8%)**
Active cardiac sarcoidosis confirmed in 7 patients Active lymphocytic myocarditis confirmed in 2 patients
**Final diagnoses of infiltrative or inflammatory cardiomyopathies**
Histologially proven sarcoidosis in 19 patients (59.4%) Presumed cardiac sarcoidosis (no biopsy performed) in 2 patients (6.3%) Lymphocytic myocarditis in 2 patients (6.3%) Cardiac amyloidosis in 1 patient (6.3%)

Abbreviations: ATTR, Amyloid Transthyretin; CMR, Cardiac Magnetic Resonance; FDG‐PET, Fluorodeoxyglucose Positron Emission Tomography; LGE, Late Gadolinium Enhancement.

CMR‐guided diagnosis prompted the initiation of immunosuppression in 23 patients (19.3% of the total cohort). Ten patients diagnosed with CS underwent implantation of a new implantable cardioverter defibrillator (ICD). Four additional patients diagnosed with CS who had an existing permanent pacemaker (PPM) at the time of diagnosis underwent an ICD upgrade because of device‐detected ventricular arrhythmias.

### 3.4 Sensitivity analysis

To evaluate the potential influence of selection bias introduced by patients with known extracardiac sarcoidosis, we conducted a sensitivity analysis excluding these individuals (*n* = 22), shown in Table S1. Among the remaining 97 patients without systemic sarcoid involvement, the prevalence of late gadolinium enhancement (LGE) remained high at 26.8% (26/97), and 14.4% (14/97) ultimately received a diagnosis of CS. These proportions were comparable to those in the full cohort (LGE in 26.9%, CS in 16.0%). Fisher's exact testing demonstrated no significant interaction between the presence of extracardiac sarcoid and either LGE detection (*p* = 1.0) or CS diagnosis (*p* = 0.34).

## 4 DISCUSSION

The central finding of the present study is that CMR‐detected myocardial fibrosis suggestive of an underlying CMP is present in approximately one‐quarter of patients with advanced conduction system disease with apparently normal LV function on echocardiography. CS was the most prevalent underlying etiology, with histologically proven sarcoidosis present in 16% of patients in this study.

A unique and more novel aspect of the present study is the inclusion of patients with various forms of conduction system disease. A number of existing studies have reported on the prevalence of infiltrative CMPs such as CS in patients with complete heart block or high‐degree atrioventricular block.^4^, ^5^, ^8^, ^9^ In this context, Heart Rhythm Society (HRS) consensus guidelines recommend using advanced cardiac imaging (CMR or PET) in patients ≤60 years old with unexplained Mobitz II or complete heart block.^10^ Conversely, there is a paucity of data addressing the prevalence of cardiac fibrosis in patients with LBBB and bifascicular block.^11^ In this study, 27% of included patients had bifascicular block, and these patients featured a similar prevalence of LGE and underlying CMP compared to patients with Mobitz II and CHB. This suggests that CMR evaluation should be considered more broadly across the spectrum of conduction system disorders.

Early diagnosis of infiltrative CMP has significant implications for clinical management. Inflammatory disorders such as sarcoidosis and lymphocytic myocarditis require prompt institution of immunosuppression to limit disease progression.^12^ Recovery of high‐degree AV block has been reported in up to 50% of patients with CS receiving early corticosteroid treatment.^13^ Immunosuppression also reduces the rate of downstream ventricular arrhythmias and promotes reverse remodeling function in patients with early‐stage sarcoidosis, although steroid efficacy is attenuated in later‐stage disease.^14^, ^15^ Diagnosis of cardiac amyloidosis may also allow for initiation of disease‐modifying amyloidosis therapy targeting either the culprit AL or ATTR protein.

Additionally, the diagnosis of CS in the setting of advanced conduction system disease has crucial implications for the choice of CIED therapy. Several studies have shown that patients with CS who present with high‐degree AV block at diagnosis are at increased risk of ensuing ventricular tachyarrhythmias and sudden cardiac death.^16^, ^17^ In this context, consensus guidelines from the HRS provide a class II recommendation for upfront ICD implantation in patients with CS and an indication for PPM therapy.^10^ In our cohort, 12% of patients underwent a change in device strategy, either upfront ICD or ICD lead upgrade, as a result of CMR evaluation. These findings support the use of CMR prior to device implantation in younger patients with intermittent AV block and preserved systolic function, to enable early detection of CS and guide appropriate device therapy.

This study also demonstrates the utility of CMR in the identification of subtle LV systolic dysfunction. Thirteen percent of patients in the present study were found to have LVEF <50% by CMR volumetric analysis that was not revealed by initial TTE imaging. CMR has increased sensitivity and lower intertest variability compared to TTE for the diagnosis of both left and right ventricular systolic function.^18^, ^19^ Identification of LV systolic dysfunction may allow for early institution of guideline‐directed medical therapy such as beta‐blockade and renin‐aldosterone‐angiotensin system inhibition.^20^

Cardiac amyloidosis has a strong association with conduction system disease.^21^ However, only one patient in this study received a final diagnosis of cardiac amyloidosis. This is likely explained by the relatively young age of the study cohort, as cardiac amyloid is most prevalent in older adults. It has also been shown that AL amyloidosis, which is the dominant form of cardiac amyloid in younger patients, is more weakly associated with conduction system disease compared to ATTR amyloid.^6^ Moreover, these patients have increased LV wall thickness on echocardiography, so it is likely the diagnosis was made by other modalities without the need for CMR. Systematic use of CMR evaluation in an older population of patients with conduction system disease may reveal a higher proportion of patients with amyloidosis.

### 4.1 Study limitations

This study has several limitations. First, this analysis only included patients who were selected to undergo CMR for the evaluation of advanced conduction system disease. This introduces inherent selection bias, as patients with the highest pre‐test probability of an underlying CMP would be most likely to be selected to undergo CMR. Further studies examining the diagnostic yield of CMR in all comers with conduction system disease are required to define the true prevalence of myocardial fibrosis and CMP in this population. Second, the diagnostic workup following initial CMR evaluation in this study was highly heterogeneous. Systematic adoption of PET or EMB in patients with CMR‐detected cardiac fibrosis may improve the detection rate of underlying CMP. Third, a small subset of patients demonstrated nonspecific LGE patterns, and while these may reflect early cardiomyopathic processes, we cannot exclude the possibility of incidental myocardial fibrosis in these cases. Finally, we only evaluated for CMR evidence of LGE, which often reflects regional fibrosis or scar. The use of tissue characterization techniques that can provide an assessment of diffuse fibrosis such as T1 mapping or extracellular volume (ECV) may also have additional utility.

## 5 CONCLUSIONS

Cardiac fibrosis is present in a substantial proportion of patients undergoing CMR for the investigation of advanced conduction system disease with apparently structurally normal hearts. CMR may be an important adjunctive tool for the investigation of conduction system disease, particularly in younger patients.

## CONFLICT OF INTEREST STATEMENT

The authors do not have any conflicts of interest to disclose.

## ETHICS STATEMENT

This study was approved by the Alfred Health Ethics Committee (22/147).

## FINANCIAL DISCLOSURES

J.W. is supported by an Australian National Heart Foundation (NHF) postgraduate scholarship. The following industry funding sources regarding activities outside the submitted work have been declared in accordance with ICMJE guidelines. P.M.K. has received funding from Abbott Medical for consultancy and speaking engagements and has served on the advisory board with fellowship support from Biosense Webster. S.P. is supported by NHMRC postdoctoral fellowship and has received fees from Abbott Medical and Biosense Webster for consultancy and speaking engagements. Other authors: No disclosures.

## PATIENT CONSENT STATEMENT

Individual patient consent was not required for this retrospective study.

## Supporting information


**Table S1.** Sensitivity analysis of patients with and without extracardiac sarcoidosis.

## Data Availability

All relevant summarized, and de‐identified data are included within the manuscript.
