# Supplementary material for: Prevalence of cardiac fibrosis and infiltrative cardiomyopathy in patients with advanced conduction system disease
Source: J Arrhythm. 2025 Jul 6;41(4):eJOA370109. doi: 10.1002/joa3.70109 (PMC12230196; doi:10.1002/joa3.70109)
Supplement: Supplementary file 1 — Table S1. Sensitivity analysis of patients with and without extracardiac sarcoidosis. [file JOA3-41-eJOA370109-s001.docx]

**Supplemental Table 1: Sensitivity analysis of patients with and without extracardiac sarcoidosis.**

| **Group** | **All patients**  **(n=119)** | **With extracardiac sarcoid (n=22)** | **Without extracardiac sarcoid (n=97)** | **Fisher’s exact p-value** |
| --- | --- | --- | --- | --- |
| **LGE positive** | 32 (26.9%) | 6 (27.3%) | 26 (26.8%) | 1.00 |
| **Final diagnosis sarcoidosis** | 19 (16.0%) | 5 (22.7%) | 14 (14.4%) | 0.34 |

**Abbreviations:** LGE, late gadolinium enhancement; CS, cardiac sarcoidosis.
